# Silver Nanostar-Based SERS for the Discrimination of Clinically Relevant *Acinetobacter baumannii* and *Klebsiella pneumoniae* Species and Clones

**DOI:** 10.3390/bios13020149

**Published:** 2023-01-17

**Authors:** Miguel Peixoto de Almeida, Carla Rodrigues, Ângela Novais, Filipa Grosso, Nicolae Leopold, Luísa Peixe, Ricardo Franco, Eulália Pereira

**Affiliations:** 1 LAQV/REQUIMTE, Departamento de Química e Bioquímica, Faculdade de Ciências, Universidade do Porto, 4169-007 Porto, Portugal; 2 UCIBIO—Applied Molecular Biosciences Unit, Department of Biological Sciences, Laboratory of Microbiology, Faculty of Pharmacy, University of Porto, 4050-313 Porto, Portugal; 3 Associate Laboratory, Institute for Health and Bioeconomy, Faculty of Pharmacy, University of Porto, 4050-313 Porto, Portugal; 4 4TOXRUN, Toxicology Research Unit, University Institute of Health Sciences, CESPU (IUCS-CESPU), 4585-116 Gandra, Portugal; 5 Faculty of Physics, Babeş-Bolyai University, 400084 Cluj-Napoca, Romania; 6 Associate Laboratory i4HB—Institute for Health and Bioeconomy, School of Science and Technology, Universidade NOVA de Lisboa, 2819-516 Caparica, Portugal; 7 UCIBIO—Applied Molecular Biosciences Unit, Departamento de Química, School of Science and Technology, Universidade NOVA de Lisboa, 2819-516 Caparica, Portugal

**Keywords:** vibrational spectroscopy, surface-enhanced Raman spectroscopy, SERS, silver nanostars, bacterial differentiation, clones, critical pathogens, chemometrics

## Abstract

The development of rapid, reliable, and low-cost methods that enable discrimination among clinically relevant bacteria is crucial, with emphasis on those listed as WHO Global Priority 1 Critical Pathogens, such as carbapenem-resistant *Acinetobacter baumannii* and carbapenem-resistant or ESBL-producing *Klebsiella pneumoniae*. To address this problem, we developed and validated a protocol of surface-enhanced Raman spectroscopy (SERS) with silver nanostars for the discrimination of *A. baumannii* and *K. pneumoniae* species, and their globally disseminated and clinically relevant antibiotic resistant clones. Isolates were characterized by mixing bacterial colonies with silver nanostars, followed by deposition on filter paper for SERS spectrum acquisition. Spectral data were processed with unsupervised and supervised multivariate data analysis methods, including principal component analysis (PCA) and partial least-squares discriminant analysis (PLSDA), respectively. Our proposed SERS procedure using silver nanostars adsorbed to the bacteria, followed by multivariate data analysis, enabled differentiation between and within species. This pilot study demonstrates the potential of SERS for the rapid discrimination of clinically relevant *A. baumannii* and *K. pneumoniae* species and clones, displaying several advantages such as the ease of silver nanostars synthesis and the possible use of a handheld spectrometer, which makes this approach ideal for point-of-care applications.

## 1. Introduction

Antimicrobial resistance (AMR) is a global health problem associated with dramatic social and economic consequences. The World Health Organization (WHO) has acknowledged this challenge and called for coordinated action, reinforcing the need for research on and the development of new antimicrobial medicines, vaccines, and diagnostic tools targeting WHO Global Priority 1 Critical Pathogens, including carbapenem-resistant *Acinetobacter baumannii* and carbapenem-resistant or ESBL-producing *Klebsiella pneumoniae* [1]. Tight epidemiological monitoring is crucial for quick interventions for the surveillance and control of hospital-acquired infections by these pathogens, though available tools are in most cases costly and time-consuming, such as matrix-assisted laser desorption/ionization time-of-flight mass spectrometry (MALDI–TOF MS) [2,3].

Vibrational spectroscopic techniques might play a role in the clinical microbiology toolbox, considering that they are fast and low-cost, and may offer point-of-care bacterial identification [4,5,6,7]. These techniques rely on the discriminatory potential of fingerprinting biochemical profiles that are obtained from pure bacterial cultures. The complexity of the chemical data captured in the spectra is usually analyzed with chemometric tools that comprise mathematical and statistical methods to interpret, model, and classify spectral variance [8]. Fourier transform infrared (FT-IR) spectroscopy and Raman spectroscopy are complementary techniques that have been more frequently used for bacterial discrimination with variable sensitivity and resolution [9,10,11,12,13]. Some of us have recently shown the ability of FT-IR spectroscopy to discriminate between and predict *A. baumannii* and *K. pneumoniae* clones with successful applications in the clinical setting [9,10,14,15].

Raman spectroscopy has also shown promising results for bacterial identification, although most of the studies included small samples and did not always involve clinical isolates belonging to the most widespread lineages [11,12,13]. Raman signals can be derived from simple molecules (e.g., metabolites) and from more complex biomolecules (e.g., proteins), and can be used for bacterial discrimination based on spectra obtained from whole or lysed bacterial samples [12,16]. However, since Raman signals have very low intensity, long acquisition times are needed to obtain satisfactory structural information, which may lead to sample degradation and featureless dispersion signals. This downside can be overcome by using surface-enhanced Raman spectroscopy (SERS) where signals from molecules adsorbed to the surface of nanostructured metals, at hot spots, can be enhanced up to 10^12^ fold. Among nanomaterials for SERS, star-shaped silver nanoparticles (AgNSs) are among the most promising nanoparticles for SERS applications. In fact, AgNSs show superior plasmonic properties and remarkable capacity to form hot spots, which makes them ideal substrates for the adsorption of Raman-active species. The multiplicity of the number or arms, arm length, and resulting global size basically enable interaction with light across all the visible spectra, although with higher intensity at the ~400 nm region [17]. Furthermore, AgNSs fulfill all the requirements regarding the typical morphology that favors hot spots, namely, rough surface, sharp tips, and intra- and interparticle nanogaps [18]. Some of us had already proven the high level of SERS performance of these AgNSs to trace analyte detection on paper substrates [19], and we had succeeded in discriminating between Portuguese wines (type and region) using SERS and chemometric methods [20]. In addition, the use of the partial least-squares discriminant analysis (PLS-DA) method combined with a SERS sensor based on silver and gold nanoparticles allowed for the effective discrimination of three foodborne pathogenic bacteria, *Staphylococcus aureus*, *Escherichia coli* and *Listeria monocytogenes,* significantly reducing the time to result in comparison with traditional analytical methods [21]. In another example, Villa et al. succeeded in differentiating between bacterial genera by using gold nanoparticle–filter paper hybrids as SERS platforms together with PLS-DA, suggesting that the obtained discrimination was probably the result of type and quantity of metabolites resulting from the purine degradation deposited in the cell wall [22]. The use of SERS for bacteria fingerprinting allows for drastically reducing the acquisition time from several minutes to a few seconds, avoiding biological sample deterioration by the laser and speeding up the overall process. Specifically, for SERS-based protocols to be competitive with well-implemented MALDI–TOF MS methods, for the rapid detection and characterization of antibiotic-resistant bacteria, two aspects need improvement: (1) SERS-active platforms that can be synthesized easily, and, most importantly, reproducibly; (2) the possibility of using a portable Raman detection system [16].

In this work, we assessed the potential of using AgNSs deposited on filter paper as SERS platforms for clinically relevant antibiotic-resistant bacterial discrimination at the species and infraspecies levels using the chemometric analysis of quickly obtained SERS spectra.

## 2. Materials and Methods

All the reagents were used as purchased with no further steps of purification. For nanoparticle synthesis, a KD Scientific KDS 200 syringe pump holding a BD 60 mL plastic syringe was used. Centrifugations were performed in a Sigma 3–30K centrifuge. Ultraviolet–visible spectroscopy (UV–vis) was performed using a Varian Cary 50 Bio spectrophotometer. Nanoparticle tracking analysis (NTA) was performed with a Malvern Nanosight NS300 equipped with a 642 nm (red) laser module via the acquisition of 5 videos of 1 min each in static mode.

### 2.1. Preparation of Silver Nanostars

Star-shaped silver nanoparticles were synthesized based on the method from Garcia-Leis et al. [17]. The following reagents were used: silver nitrate 99.9999% (Aldrich, St. Louis, MO, USA), hydroxylamine solution 50% wt. in water 99.999% (Aldrich), sodium hydroxide 98% (Fisher, Waltham, MA, USA), and trisodium citrate dihydrate 99.0% (Merck, Darmstadt, Germany). All glassware was previously treated with aqua regia and rinsed abundantly with deionized water, followed by ultrapure water (18.2 MΩ·cm at 25 °C). All solutions were prepared in ultrapure water. For the synthesis reaction, 2.5 mL of a 50 mmol·dm^−3^ sodium hydroxide solution and 2.5 mL of a 60 mmol·dm^−3^ hydroxylamine solution were mixed in beaker, and 45 mL of a 1 mmol·dm^−3^ silver nitrate solution was added dropwise from a syringe immediately afterwards, using a syringe pump at a 45 mL/min injection rate. After 90 s, 500 µL of a 1.5% wt. trisodium citrate dihydrate solution was added to the mixture. The reaction vessel was kept in the dark for 3 h. Then, the content of the five beakers was mixed, and the resultant batch (250 mL) of AgNS suspension was centrifuged for 12 min at a relative centrifugal force (RCF) of 1600 g. The pellet was resuspended in ultrapure water (up to 10% of the initial volume) and stored in glass vials.

### 2.2. Raman and SERS Experiments

Raman and SERS spectra were measured with a Renishaw InVia Raman microscope coupled with 633 nm 17 mW (He–Ne) and 785 nm 300 mW (diode) lasers. Gratings of 1200 and 1800 L/mm were used, the first for 785 nm measurements and the second for 633 nm. The system was equipped with a CCD camera detector with a 1040 × 256 resolution. The used objective had 50× magnification. In parts of this work, a Labram 300 Horiba Jobin Yvon spectrometer equipped with a laser of 633 nm (He–Ne), focused with a 50× objective, was used.

### 2.3. Bacterial Strains

We selected 58 well-characterized multidrug-resistant *A. baumannii* and *K. pneumoniae* isolates from clinical origin to validate the approach. *A. baumannii* isolates represent the main clones (ST98 (*n* = 4); ST103 (*n* = 1); ST208 (*n* = 4) and ST218 (*n* = 2), ST515 (*n* = 1) and ST1557 (*n* = 1)) circulating in Portugal between 2001 and 2015, exhibiting multidrug- and extensive resistance phenotypes together with variable capsule types [9]. *K. pneumoniae* strains, representing clinically relevant and globally disseminated clones, were used to assess interspecies and intraspecies discrimination. They comprised diverse ST11 (*n* = 7), ST14 (*n* = 5), ST15 (*n* = 14), ST101 (*n* = 5), ST147 (*n* = 8), and ST258 (*n* = 6) carrying different capsular types and identified along time [10].

Clonal relatedness between isolates had previously been established with reference genotypic methods (pulsed-field gel electrophoresis, multilocus sequence typing) [9,10]. Details about the bacterial isolates included in this study are summarized in Table S1 (Supplementary Materials).

### 2.4. Preparation of the SERS Samples

Bacterial strains were grown on Mueller–Hinton agar at 37 °C for 18 h to reach the stationary growth phase. A 1 µL loop of pure bacterial cells was mixed and manually homogenized with 20 µL of a 0.5 nmol·dm^−3^ silver nanostar suspension, immediately deposited on filter paper (Whatman 2), and dried at room temperature. Microphotographs of the deposited mixtures are presented in Figure S1 (Supplementary Materials). For each isolate, at least 21 instrumental replicates (obtained from the same bacterial nanoparticle suspension) and 3 biological replicates (obtained in three independent days) were acquired and analyzed, corresponding to a minimum of 9 spectra per strain. Filter papers were incinerated immediately after measurements.

### 2.5. SERS Measurements

Raman spectra were acquired in a 7 × 3 matrix from a total of 21 different and nonoverlapping spots (with Renishaw equipment). For the extended experiments with *A. baumannii* and *K. pneumoniae*, at least 3 spectra were manually (not as the preset matrix) acquired (with Horiba equipment). Acquisition times varied from 4 to 20 s, depending on the laser and Raman microscope.

### 2.6. SEM Characterization

SEM observations were carried out using a Carl Zeiss AURIGA CrossBeam Workstation. Samples (filter paper with dry suspension) were placed on the SEM support using carbon conductive double-sided adhesive tape.

### 2.7. Spectral Data Analysis

Raman chemometric analyses were performed using MATLAB R2015a version 8.5 (MathWorks, Natick, MA, USA) and PLS Toolbox version 8.5 for MATLAB (Eigenvector Research, Manson, WA, USA). Original Raman spectra were processed to minimize variation related with spectral acquisition, and to amplify differences in the spectra acquired in similar conditions to facilitate the subsequent interpretation and analysis. We used the Savitzky–Golay filter (filter = 7; order = 5; first or second derivative order), smoothing (filter = 5; order = 2), and normalization, following previous suggestions [23,24]. Instrumental replicates for the same bacterial culture/suspension were average for simplification.

Pattern recognition methods were used to interpret spectral diversity using whole spectra. We used principal component analysis (PCA), an unsupervised method that allows for capturing spectral diversity without an a priori class assignment, where variation is captured in the principal components (PCs) and expressed in score plots. Partial least-squares discriminant analysis (PLSDA) was the supervised machine-learning method used to build classification models based on the *a priori* knowledge of the sample and classes (sequence types, capsule composition) to extract specific spectral signatures. The number of latent variables (LVs) was optimized using the leave-one-sample-out cross-validation procedure to prevent overfitting as previously [10].

## 3. Results

**Figure 1:**
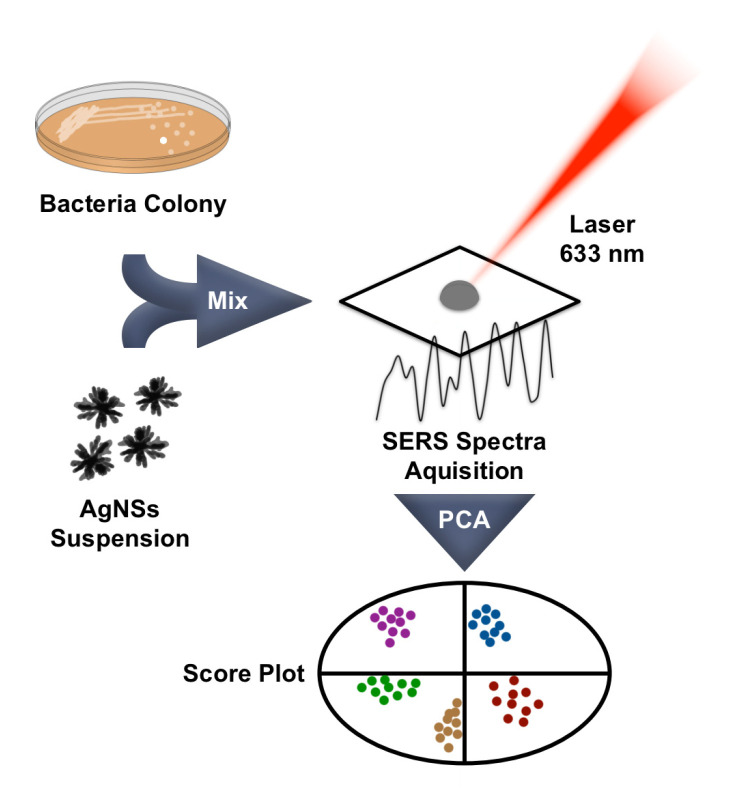
Schematic representation of the protocol for bacterial discrimination.

Figure 1 describes our approach towards the discrimination of clinically relevant bacterial species and lineage. The optimized protocol is extremely straightforward, as SERS measurements are performed in a mixture of a AgNS suspensions with a bacterial colony that is deposited on a filter paper. After acquisition, data are treated using multivariate data analysis tools.

### 3.1. Optimization of the Experimental Protocol

Several experimental parameters, such as the capping of AgNSs, the composition of the media where AgNS bacterial colony incubation was performed, incubation time, biomaterial amount, and laser wavelength, were initially assessed. The final protocol to be used was selected based on the best results for optimal naked-eye AgNS–bacterial distribution, the intensity and quality of the SERS signal, and the capacity for bacterial discrimination (data not shown). Regarding the capping of AgNSs, two capping agents were tested: cysteamine, which chemisorbs to the silver surface with slow exchanging rates, and citrate, a capping agent that physiosorbs to the silver surface, showing a much faster exchange with other molecules in solution. Citrate gave better results, which indicates that, upon the incubation of AgNSs with the cells, the fast exchange of the capping agent with molecules from the bacterial wall or the media provided better discrimination. In the media in which AgNSs-bacterial colony incubation was performed, ultrapure water and phosphate buffer saline were tested, with the experiments using ultrapure water giving better results. The incubation times of 1, 10, and 30 min were examined with no appreciable variation in the obtained results. The shorter incubation time of 1 min was then selected. Regarding the most appropriate laser wavelength, the green (532 nm) laser was excluded due to fluorescence interference. Conversely, the red (633 nm) and infrared (785 nm) lasers were used down to intraspecies discrimination since they have lower fluorescence interferences. Infrared was a better choice to avoid fluorescence phenomena; however, probably due to the higher SERS enhancement effect at 633 nm compared to that at 785 nm, linked with weaker scattering at 785 nm compared to that at 633 nm, the results obtained using the red laser (633 nm), provided better discrimination. The optimized protocol is presented in Table 1, and it was used in all experiments for bacterial differentiation.

**Figure 2:**
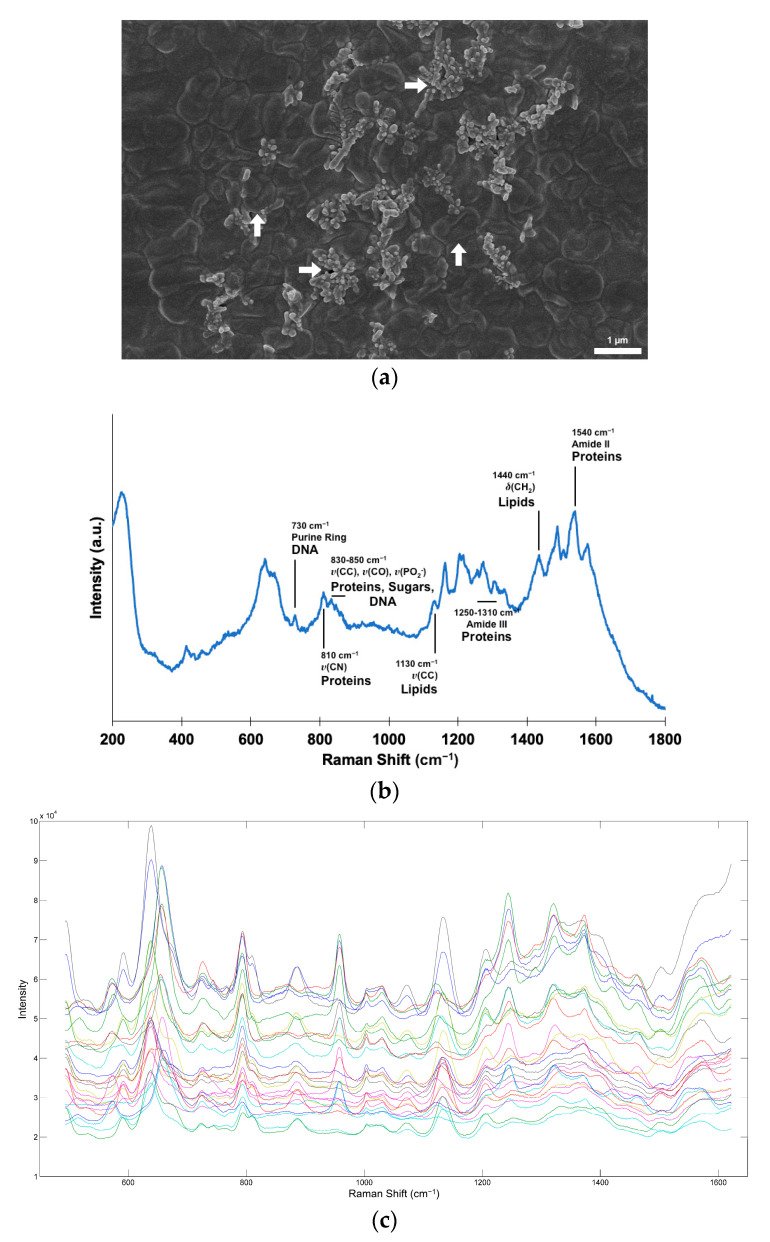
(**a**) SEM micrograph of *A. baumannii* incubated with AgNSs (10,000× magnification). AgNSs are indicated by horizontal arrows, and bacterial cells are indicated by vertical arrows; (**b**) corresponding SERS spectrum with tentative attributions of biomolecules to some of the vibrational lines; (**c**) SERS spectra of multiple *A. baumannii* isolates.

Figure 2a shows a SEM micrograph obtained from a colony of *K. pneumoniae* incubated with AgNSs according to the optimized protocol. AgNSs (indicated by horizontal arrows) could be detected in close contact with bacterial cells (indicated by vertical arrows). Figure 2b presents the corresponding SERS spectrum. The main detected vibrational lines could be associated with sugars, lipids, proteins, and DNA [25,26,27,28,29]. Figure 2c shows *A. baumannii* Raman spectra prior to preprocessing, where it is possible to visualize differences in spectra from different isolates, with the major peaks at 600–700, 800, 950, and 1150 cm^−1^, which is consistent with previous studies [25,26,27].

### 3.2. Discrimination between A. baumannii and K. pneumoniae

**Figure 3:**
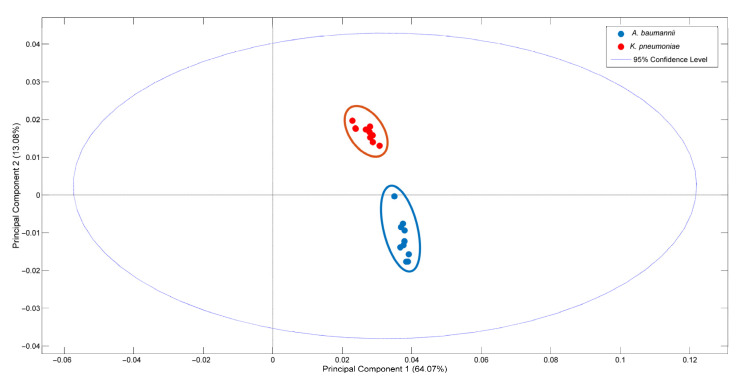
Discrimination of *K. pneumoniae* and *A. baumannii* species via SERS. Score plot of PCA model with the first two principal components (PCs) showing the differentiation of Raman spectra according to species.

We tested the ability of SERS coupled with chemometrics to differentiate between *A. baumannii* and *K. pneumoniae*, two of the most clinically relevant species. We used 10 isolates per species that represented diverse clinically relevant clones from each species (see Section 2 for details). The score plot obtained with PCA shows that spectra from each species were grouped in two well-delimited clusters, with PC1 capturing around 64% of the spectral variance (Figure 3). The loadings of the model show that spectral regions 650–680, 720, 930–970, and 1230–1350 cm^−1^ contributed the most for the discrimination between these species (data not shown).

### 3.3. Discrimination of A. baumannii and K. pneumoniae at the Infraspecies Level

**Figure 4:**
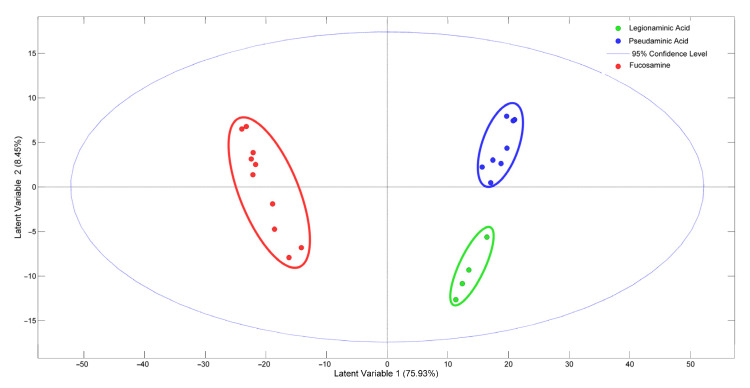
Discrimination of *A. baumannii* clones by SERS. Score plot of the PLSDA classification model with the first two latent variables (LVs) showing the differentiation of Raman spectra according to the capsule composition (fucosamine, legionaminic acid, pseudaminic acid).

To assess the discriminatory ability of SERS-enhanced Raman spectra to differentiate within *A. baumannii* and *K. pneumoniae* species (i.e., at the intraspecies level), we tested isolates representing different well-defined clones of each of these species (see Supplementary Table S1). *A. baumannii* isolates belonging to widely disseminated clones (ST98, ST103, ST208, and ST218) were tested, and the score plot of the supervised PLSDA shows that clusters were formed by isolates displaying the same capsule sugar composition (Figure 4). Although ST98 and ST103 are unrelated clones, they group together, which may be explained by the fact that they present the same capsule locus (KL9) predicted by whole-genome sequencing analysis, which corresponds to fucosamine composition. In addition, ST208 (KL2), which displays a capsule with pseudaminic acid, and ST218 (KL7), which displays a capsule with legionaminic acid, formed independent clusters (Figure 4, Supplementary Table S2) [9]. The specificity (0.93–1.0) and sensitivity (0.75–1.0) of the model were high despite the small sample (Supplementary Table S2).

**Figure 5:**
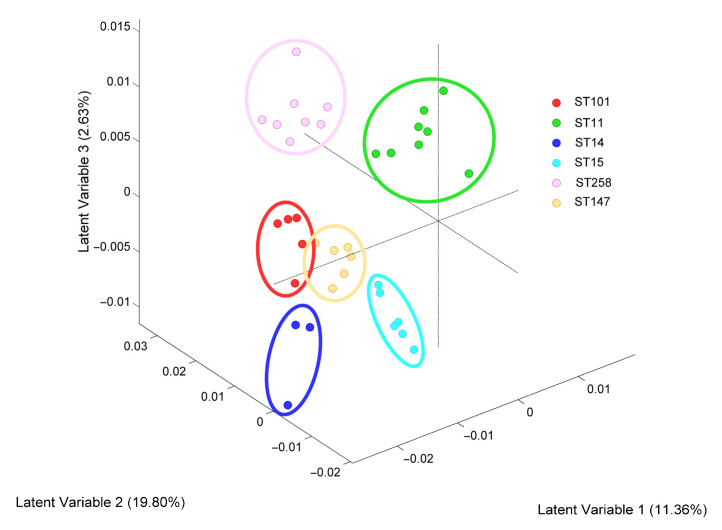
Discrimination of *K. pneumoniae* clones by SERS. Score plot of the PLSDA classification model with three latent variables (LVs) showing differentiation of Raman spectra according to the sequence type (ST).

To assess the ability to differentiate between *K. pneumoniae* clones, isolates belonging to clinically relevant clones (ST11, ST14, ST15, ST101, ST147, ST258) were tested in the same experimental conditions. The score plot of the supervised PLSDA shows that isolates belonging to each clonal type were grouped independently, demonstrating a characteristic biochemical composition for each clone (ST) that was differentiated with SERS (Figure 5). The specificity of the model was very high (0.73–1.0), while sensitivity should be improved with a larger and more proportional sample per class (Supplementary Table S3).

PLSDA model loadings reveal that discrimination at the intraspecies level was mainly due to a combination of spectral peaks at 570–660, 950, 1130, and 1250 cm^−1^.

## 4. Discussion

In this work, SERS measurements of bacterial colonies incubated with AgNSs were explored as a technique capable of differentiating between species considered to be critical pathogens by the WHO and clinically relevant antibiotic resistant clones [9,10,30]. The results obtained in this pilot study demonstrate that the biochemical information contained in SERS spectra obtained from pure bacterial cultures can be used to establish discriminatory bacterial fingerprinting profiles, as observed previously for other vibrational spectroscopic techniques [4,31,32,33]. Considering previous studies attempting to assign Raman peaks to different biomolecules [34], proteins (1130 cm^−1^) may be important for discrimination at the intraspecies level, while proteins and carbohydrates (1230–1350 cm^−1^), and adenine (720 cm^−1^), a typical metabolite of purine degradation, seem to contribute to species differentiation.

In previous studies, SERS was used to distinguish species from different genera or to differentiate antibiotic-resistant from antibiotic-susceptible bacteria [16,35]. Additionally, SERS was used to differentiate between *A. baumannii* [12] or *K. pneumoniae* clones [13], though only specific reference isolates were tested, and correlation with bacterial features was mostly lacking. With our SERS protocol and chemometric analysis, we were able to establish for the first time that *A. baumannii* discrimination is correlated with capsule composition, similarly to what was observed for FTIR spectroscopy [9]. Conversely, *K. pneumoniae* clones were discriminated based on clones defined by ST, suggesting that other biochemical features contribute to discrimination.

Two recent reviews on SERS-based methods for discriminating between antibiotic-resistant bacteria describe different combinations of nanostructured materials, bacterial sample preparation steps, and labeling schemes [16,35]. Nevertheless, none of the described examples is as simple and easy to use as our system, at three levels: (i) we used AgNSs, which are very easy to synthesize in a reproducible manner by following a well-established protocol [17]. Furthermore, the best results were obtained with the as-synthesized AgNSs, with no further functionalization. (ii) No bacterial sample processing is involved: a bacterial suspension derived from a single colony, is mixed with the AgNSs-containing solution immediately before SERS analysis. (iii) Both bacteria and AgNSs are unlabeled. After AgNSs–bacteria incubation, they are deposited on filter paper for SERS spectral acquisition, and spectral data are analyzed using chemometric tools, as previously described [20]. This is an easy-to-perform protocol, providing quick results (less than 30 min from sample deposition to result output) after the overnight obtention of isolated bacterial colonies. The use of Raman spectroscopy instead of gold-standard methods (e.g., mass spectrometry or whole-genome sequencing) lowers the cost of the equipment needed for analysis and brings the possibility to perform point-of-care diagnostics [36]. Moreover, the pathogenic bacteria-containing filter paper can be easily incinerated for appropriate disposal.

Additional studies with larger samples, including a higher diversity of isolates per species and a higher number of different Gram-negative and Gram-positive species, are required to validate this approach and the reproducibility of the experimental conditions. However, the protocol and results established here, together with the results from previous studies, are a proof of principle for SERS-based bacterial discrimination, and might guide future experiments to expand this application, and optimize experimental conditions and analytical models.

## Figures and Tables

**Table 1:** Parameters and respective values for the optimized SERS–AgNS protocol.

Parameter	Value
Sample volume	20 µL (dry)
SERS material	Silver (nanostars)
Raman system	Confocal (50× objective)
Laser	633 nm (He–Ne)
Spectral resolution	1 cm^−1^
Focal spot area	1 µm^2^
Acquisition time	2 × 10 s
Spectral range	200–1800 cm^−1^
Baseline correction	None

## Data Availability

Data are presented in the article. Initial instrumental output data are available upon request from the corresponding author (RF).

## Supplementary Materials

The following supporting information can be downloaded at: https://www.mdpi.com/article/10.3390/bios13020149/s1. Table S1: details about the bacterial isolates included in this study; Table S2: statistics of the PLSDA model for the discrimination of *A. baumannii* clones (Figure 4); Table S3: statistics of the PLSDA model for the discrimination of *K. pneumoniae* clones (Figure 5); Figure S1: micrographs of (left) the original filter paper support and (right) the spot with the sample—50× magnification.
