# Supplementary material for: Silver Nanostar-Based SERS for the Discrimination of Clinically Relevant Acinetobacter baumannii and Klebsiella pneumoniae Species and Clones
Source: Biosensors (Basel). 2023 Jan 17;13(2):149. doi: 10.3390/bios13020149 (PMC9953856; doi:10.3390/bios13020149)
Supplement: Supplementary file 1 [file biosensors-13-00149-s001.zip › biosensors-2142253-supplementary.pdf]

# Supplementary Materials

## Silver nanostars-based SERS for discrimination of clinically relevant *Acinetobacter baumannii* and *Klebsiella pneumoniae* species and clones

Miguel Peixoto de Almeida, Carla Rodrigues, Ângela Novais, Filipa Grosso, Nicolae Leopold, Luísa Peixe, Ricardo Franco and Eulália Pereira

**Table S1.** Details about the bacterial isolates included in this study.

<table>
<tr><th>Species</th><th>Sequence Type</th><th>Isolate</th><th>Year of Isolation</th><th>Capsular type (capsule locus - KL)</th></tr>
<tr><td rowspan="13">A. baumannii</td><td rowspan="4">98</td><td>Ac2</td><td>2001</td><td rowspan="4">fucosamine · KL9</td></tr>
<tr><td>Ac25</td><td>2006</td></tr>
<tr><td>Ac32</td><td>2001</td></tr>
<tr><td>Ac55</td><td>2002</td></tr>
<tr><td>ST103</td><td>Ac70</td><td>2002</td><td></td></tr>
<tr><td rowspan="4">208</td><td>HPH1</td><td>2006</td><td rowspan="4">pseudaminic acid · KL2</td></tr>
<tr><td>H466</td><td>2011</td></tr>
<tr><td>H580</td><td>2012</td></tr>
<tr><td>H678</td><td>2013</td></tr>
<tr><td rowspan="2">218</td><td>H202</td><td>2010</td><td rowspan="2">legionaminic acid · KL7</td></tr>
<tr><td>H631</td><td>2013</td></tr>
<tr><td>515</td><td>H181</td><td>2010</td><td></td></tr>
<tr><td>1557</td><td>316.1</td><td>2015</td><td>pseudaminic acid · KL2</td></tr>
<tr><td rowspan="13">K. pneumoniae</td><td rowspan="13">ST15</td><td>C1699</td><td>2012</td><td>KL112</td></tr>
<tr><td>44</td><td>2013</td><td>KL24</td></tr>
<tr><td>C1686</td><td>2012</td><td>KL24</td></tr>
<tr><td>C1693</td><td>2012</td><td>KL24</td></tr>
<tr><td>K47</td><td>2012</td><td>KL110</td></tr>
<tr><td>C1694</td><td>2012</td><td>KL19</td></tr>
<tr><td>H1144</td><td>2010</td><td>KL112</td></tr>
<tr><td>H1119</td><td>2010</td><td>KL24</td></tr>
<tr><td>H1111</td><td>2010</td><td>KL48</td></tr>
<tr><td>H1120</td><td>2010</td><td>KL24</td></tr>
<tr><td>C1733</td><td>2012</td><td>KL19</td></tr>
<tr><td>K88</td><td>2015</td><td>KL19</td></tr>
<tr><td>K95</td><td>2015</td><td>KL19</td></tr>
</table>

| | | | |
|---|---|---|---|
| | K43 | 2011 | KL110 |
| ST14 | H49 | 2003 | KL2 |
| | H1122 | 2010 | KL16 |
| | H55 | 2002 | KL2 |
| | H153 | 2003 | K2 |
| | H39 | 2002 | KL16 |
| ST147 | K87 | 2015 | KL64 |
| | K89 | 2015 | KL64 |
| | K7 | 2014 | KL64 |
| | K105 | 2015 | KL64 |
| | C1716 | 2012 | KL64 |
| | 10D79 | 2010 | KL64 |
| | H1134 | 2010 | KL64 |
| | H669 | 2006 | KL64 |
| ST11 | C1721 | 2012 | KL105 |
| | 13I15 | 2012 | KL24 |
| | 10E34 | 2010 | KL24 |
| | 12F64 | 2012 | KL24 |
| | RP29 | 2012 | KL27 |
| | B40U | 2012 | KL64 |
| | B08U | 2012 | KL64 |
| ST258 | 2934/08 | 2008 | KL107 |
| | CRE01Kp | 2008 | KL106 |
| | CRE38 | 2006 | KL106 |
| | 5586/09 | 209 | KL106 |
| | Kp1810 | 2007 | KL106 |
| | Kp1652 | 2007 | KL106 |
| ST101 | 18 | 2012 | KL17 |
| | E21 | 2012 | KL17 |
| | RP50 | 2012 | KL17 |
| | SC26 | 2012 | KL17 |
| | B23U | 2012 | KL17 |

**Table S2.** Statistics of the PLSDA Model for discrimination of *A. baumannii* clones (Figure 4).

| **Confusion Matrix (CV)** | | | | |
|---|---|---|---|---|
| Class | TP | FP | TN | FN |
| 1 | 1.00000 | 0.00000 | 1.00000 | 0.00000 |
| 2 | 0.75000 | 0.00000 | 1.00000 | 0.25000 |
| 3 | 1.00000 | 0.07143 | 0.92857 | 0.00000 |
| **Confusion Table (CV)** | | | | |
| | | Actual Class | | |
| | | Class 1 | Class 2 | Class 3 |
| Predicted Class | Class 1 | 10 | 0 | 0 |
| | Class 2 | 0 | 3 | 0 |
| | Class 3 | 0 | 1 | 8 |
| | | Class 1 | Class 2 | Class 3 |
| Sensitivity (CV) | | 1.000 | 0.750 | 1.000 |
| Specificity (CV) | | 1.000 | 0.944 | 0.929 |
| Class. Err (Cal) | | 0 | 0 | 0 |
| Class. Err (CV) | | 0 | 0.152778 | 0.0357143 |
| RMSEC | | 0.079396 | 0.23396 | 0.184047 |
| RMSECV | | 0.0967994 | 0.300902 | 0.237687 |
| Bias | | $-5.55112 \times 10^{-17}$ | $-2.77556 \times 10^{-17}$ | $-1.66533 \times 10^{-16}$ |
| CV Bias | | 0.000375012 | -0.00894944 | 0.00857443 |
| $R^2$ Cal | | 0.974575 | 0.632045 | 0.853619 |
| $R^2$ CV | | 0.962234 | 0.400875 | 0.756911 |
| **Percent Variance Captured by Regression Model** | | | | |
| | X Block | | Y Block | |
| Component | This | Total | This | Total |
| 1 | 75.93 | 75.93 | 55.04 | 55.04 |
| 2 | 8.45 | 84.37 | 26.96 | 82.01 |

**Notes:**

Class 1: Fucosamine; Class 2: Legionaminic Acid; Class 3: Pseudaminic Acid

TP = True positives; FP = False positives; TN = True negatives; FN = False negatives

**Table S3.** Statistics of the PLSDA Model for discrimination of *K. pneumoniae* clones (Figure 5).

| Modeled Class | 11 | 14 | 15 | 101 | 147 | 258 |
|---|---|---|---|---|---|---|
| Sensitivity (Cal) | 1.000 | 1.000 | 1.000 | 1.000 | 1.000 | 1.000 |
| Specificity (Cal) | 1.000 | 1.000 | 1.000 | 1.000 | 1.000 | 1.000 |
| Sensitivity (CV) | 0.000 | 0.000 | 0.375 | 0.200 | 0.000 | 0.000 |
| Specificity (CV) | 1.000 | 1.000 | 0.731 | 0.828 | 1.000 | 0.931 |
| Class. Err (Cal) | 0 | 0 | 0 | 0 | 0 | 0 |
| Class. Err (CV) | 0.5 | 0.5 | 0.447115 | 0.486207 | 0.5 | 0.534483 |
| RMSEC | 0.104073 | 0.0936525 | 0.135531 | 0.121834 | 0.106992 | 0.12756 |
| RMSECV | 0.399505 | 0.30931 | 0.452085 | 0.375605 | 0.42603 | 0.412767 |
| Bias | 0.0091042 | 0.00305393 | 0.00729192 | 0.00133489 | -0.00337821 | 0.000801676 |
| CV Bias | 0.0508164 | -0.0061827 | 0.0276773 | 0.0449046 | -0.0291494 | 0.0135666 |
| $R^2$ Cal | 0.93426 | 0.891094 | 0.898208 | 0.881676 | 0.92131 | 0.870281 |
| $R^2$ CV | 0.0430872 | 0.0202972 | 0.0161336 | 0.0093623 | 0.0787175 | 0.157024 |

**Percent Variance Captured by Regression Model**

| | X Block | | Y Block | |
|---|---|---|---|---|
| Component | This | Total | This | Total |
| 1 | 13.81 | 13.81 | 13.23 | 13.23 |
| 2 | 18.29 | 32.10 | 9.38 | 22.61 |
| 3 | 3.29 | 35.40 | 18.44 | 41.05 |
| 4 | 3.29 | 38.69 | 16.59 | 57.64 |
| 5 | 2.52 | 41.21 | 16.84 | 74.48 |
| 6 | 4.26 | 45.47 | 15.45 | 89.93 |

Nanobioconjugates, independently of the preparation method, assured a very dark and localized spot on the support. Optical microscopy showed a fully covered support, as depicted in Figure S1.

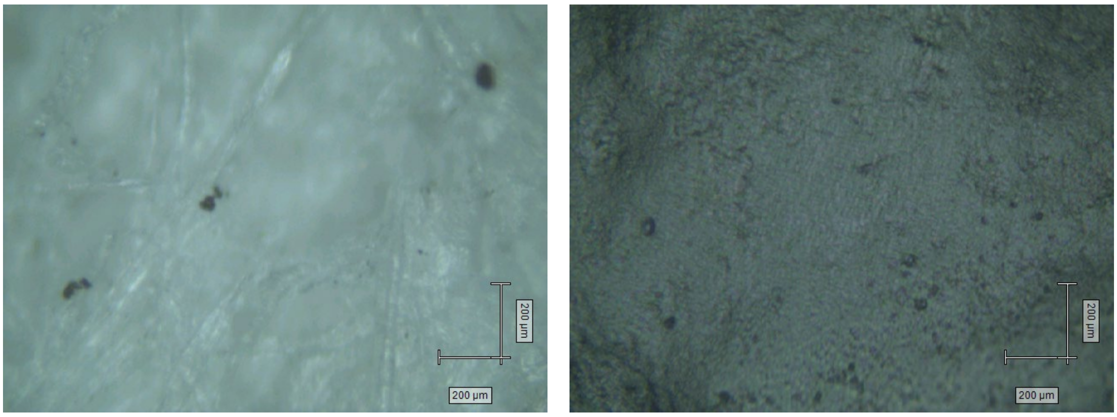


Figure S1. Micrographs of the original filter paper support (left) and of the spot with the sample (right) – 50× magnification.
